# NURR1‐deficient mice have age‐ and sex‐specific behavioral phenotypes

**DOI:** 10.1002/jnr.25067

**Published:** 2022-05-20

**Authors:** Francesca Montarolo, Serena Martire, Francesco Chiara, Sarah Allegra, Silvia De Francia, Eriola Hoxha, Filippo Tempia, Marco Alfonso Capobianco, Antonio Bertolotto

**Affiliations:** ^1^ Neuroscience Institute Cavalieri Ottolenghi (NICO) Orbassano Italy; ^2^ Neurology Department and Regional Referring Center of Multiple Sclerosis (CReSM) University Hospital San Luigi Gonzaga Orbassano Italy; ^3^ Department of Molecular Biotechnology and Health Sciences University of Turin Turin Italy; ^4^ Department of Biological and Clinical Sciences University of Turin, AOU San Luigi Gonzaga Orbassano Italy; ^5^ Department of Neuroscience “Rita Levi Montalcini” University of Turin Turin Italy

**Keywords:** dopamine, locomotion, motor impairment, murine model, NURR1, Parkinson's disease

## Abstract

The transcription factor NURR1 is essential to the generation and maintenance of midbrain dopaminergic (mDA) neurons and its deregulation is involved in the development of dopamine (DA)‐associated brain disorders, such as Parkinson's disease (PD). The old male NURR1 heterozygous knockout (NURR1‐KO) mouse has been proposed as a model of PD due to its altered motor performance that was, however, not confirmed in a subsequent study. Based on these controversial results, we explored the effects of the NURR1 deficiency on locomotor activity, motor coordination, brain and plasma DA levels, blood pressure and heart rate of old mice, also focusing on the potential effect of sex. As a probable consequence of the role of NURR1 in DA pathway, we observed that the old NURR1‐KO mouse is characterized by motor impairment, and increased brain DA level and heart rate, independently from sex. However, we also observed an alteration in spontaneous locomotor activity that only affects males. In conclusion, NURR1 deficiency triggers sex‐ and age‐specific alterations of behavioral responses, of DA levels and cardiovascular abnormalities. Further studies in simplified systems will be necessary to dissect the mechanism underlying these observations.

## Graphical abstract

NURR1 regulates the production of dopamine. Dopamine is present in dopaminergic neurons to regulate movement and emotion and in blood to control cardiac functions. Here, we demonstrated motor impairment, increased brain DA level and heart rate in old NURR1‐deficient mice independently from sex, although sex affects their spontaneous locomotion.

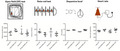

**Significance**

NURR1 is a transcription factor able to regulate the production of dopamine (DA). In brain, DA is mainly present in midbrain dopaminergic (mDA) neurons which control voluntary movement and emotion. Alteration to mDA is implicated in neurological/psychiatric disorders, including Parkinson's disease, schizophrenia, attention deficit hyperactivity disorder, and drug addiction. In periphery, DA prominently contributes to the control of cardiac and vascular functions. Here, we found that NURR1 deficiency in mouse influences the behavioral phenotype, the DA level, and the cardiovascular system in an age‐ and sex‐specific manner. In addition to clarifying the role of NURR1 in the DA pathway, the present work enhances the contribution of sex and age to the development of DA‐associated diseases.

## 1 INTRODUCTION

The midbrain dopaminergic (mDA) neurons are a class of ventral mesencephalic neurons critical for controlling voluntary movement, emotion, reward, and motivating behavior (Bissonette & Roesch, 2016). Alteration to this neuronal population is implicated in several neurological and psychiatric disorders, including Parkinson's disease (PD), schizophrenia, attention deficit hyperactivity disorder (ADHD), and drug addiction (Buervenich et al., 2000; Jankovic et al., 2005; Samaha et al., 2021; Xing et al., 2006).

The nuclear receptor‐related 1 protein (NURR1, also called NR4A2) is a transcription factor essential for the development and functioning of the dopaminergic circuitry. Specifically, NURR1 is required for mDA generation, as its ablation leads to their full agenesis (Saucedo‐Cardenas et al., 1998; Zetterström et al., 1997). NURR1 also exerts a role in the migration and target area innervation of differentiating mDA in the striatum (Solomin et al., 1999). In mature mDA, NURR1 regulates genes of the dopamine (DA) signaling pathway, such as tyrosine hydroxylase (TH), DA transporter 1 (DAT1), and vesicular monoamine transporter 2 (VMAT2) (Kadkhodaei et al., 2009; Saucedo‐Cardenas et al., 1998; Smidt & Burbach, 2007). Finally, NURR1 exerts an anti‐inflammatory function in microglia, which protects mDA from inflammation‐induced death (Kadkhodaei et al., 2013; Saijo et al., 2009). NURR1 level in mDA neurons is known to be decreased in the elderly (Chu et al., 2002), and in DA‐associated brain disorders, including PD (Jankovic et al., 2005) and schizophrenia (Buervenich et al., 2000; Xing et al., 2006). In particular, it has been reported that polymorphisms and mutations resulting in reduced expression of NURR1 are associated with familial and sporadic PD (Hering et al., 2004; Le et al., 2003; Zhang et al., 2015; Zheng et al., 2003). Recently, an increasing number of studies has provided promising results on the effect of NURR1 activation and gene delivery in vitro and in vivo in PD models, which are able to protect mDA neurons from neurotoxicity and motor behaviors associated with DA neurotransmission (De Miranda et al., 2015; Hammond et al., 2015; Oh et al., 2015). Besides its role in central nervous system (CNS), NURR1 is an active player and a potential peripheral biomarker of PD, since downregulated gene expression levels were found in peripheral blood mononuclear cells (Le et al., 2008; Liu et al., 2012) and whole blood (Montarolo et al., 2016) of PD patients. However, the implications of this peripheral downregulation have not yet been elucidated. It is known that peripheral DA contributes to the control of cardiac and vascular function, including heart rate and blood pressure (Goldberg, 1984; Ziegler et al., 1985), but possible cardiovascular effects of NURR1 mediated by DA have not yet been investigated.

In the past, the NURR1 knockout (NURR1‐KO) mouse was extensively studied. Notably, since homozygous NURR1‐KO mice die within 12 h after birth (Saucedo‐Cardenas et al., 1997, 1998), all studies were performed with heterozygous NURR1‐KO mice. The heterozygous NURR1‐KO mouse was suggested as a model for DA‐associated brain disorders, including PD (Jiang et al., 2005) and schizophrenia (Rojas et al., 2007), but further studies highlighted its restricted behavioral phenotype (Vuillermot et al., 2011). In fact, only the increased spontaneous locomotor activity in a novel environment has been observed in independent laboratories and using different protocols and NURR1‐KO models (Eells et al., 2002; Jiang et al., 2005; Rojas et al., 2007; Vuillermot et al., 2011, 2012). Starting from these considerations, we previously replicated the already highlighted altered behavioral phenotype of young male NURR1‐KO mice using a wide‐ranging test battery (Montarolo et al., 2019). As a result, we confirmed their hyperactive phenotype and we described for the first time their impulsive behavior, while we did not observe alterations in motor coordination, anxiety, sociability, and memory (Montarolo et al., 2019).

Unlike the young, only two studies reporting conflicting results investigated the phenotype of old NURR1‐KO mice (Jiang et al., 2005; Kummari et al., 2017). Jiang and colleagues observed significant alteration in both motor and locomotor activities in old NURR1‐KO mice measured by means of rotarod and open field (OF) test, respectively (Jiang et al., 2005). This was not confirmed by Kummari and colleagues, who described no significant differences in rotarod performance and OF activity in old NURR1‐KO mice in comparison to their wild‐type (WT) littermates (Kummari et al., 2017).

Aimed at elucidating whether the old NURR1‐KO mouse is a suitable behavioral model of PD characterized by motor impairment, here we examined the effects of the constitutive deletion of NURR1 on locomotor activity, motor coordination, DA levels in brain and plasma, blood pressure and heart rate of old mice. To date studies on NURR1‐KO mice, in elderly as well as in youth, have been carried out on groups of male mice only or on mixed groups of males and females. Therefore, we also explored the potential effect of sex on the behavioral phenotype of old mice.

## 2 MATERIALS AND METHODS

### 2.1 Animals

The NURR1 knockout (NURR1‐KO) mice were obtained from Prof. Orla M. Conneely, Baylor College of Medicine, Houston, USA. Since homozygous NURR1‐KO mice die within 12 h after birth (Saucedo‐Cardenas et al., 1997, 1998), heterozygous mice were used. Their genotype was confirmed by means of polymerase chain reaction (PCR) (Saucedo‐Cardenas et al., 1998). All experimental procedures were carried out at the Neuroscience Institute Cavalieri Ottolenghi (NICO), approved by the Ethical Committee of the University of Torino and authorized by the Italian Ministry of Health (authorization numbers: 56/2017‐PR and 586/2020‐PR). The experiments have been carried out in accordance with the European Communities Parliament and Council Directives of 24 November 1986 (86/609/EEC) and 22 September 2010 (2010/63/EU). Mice were housed with a 12 h light/dark cycle and free access to food/water. Adequate measures were taken to minimize pain and discomfort.

### 2.2 Behavioral tests

The WT and NURR1‐KO animals underwent the behavioral tests always during the light phase of the cycle, with at least 1 week break between tests, as reported in (Montarolo et al., 2019). At the end of each trial, the equipment was accurately cleaned with ethanol 2% and water.

### 2.3 Open field (OF) test

Locomotor activity was investigated by means of the OF test in 16‐month‐old NURR1‐KO (male *n* = 5; female *n* = 9) and their WT littermates (male *n* = 6; female *n* = 5) and in 3–5‐month‐old female NURR1‐KO (*n* = 9) and their WT littermates (*n* = 11). On test day, mice were transported to the testing room and left undisturbed for 1 h before testing. The experiments were performed under dim white light conditions (2 lux). Each animal was placed in the corner of the arena (50 × 50 × 50 cm) for 1 h. Total distance and distance traveled in the center (25 × 25 cm) were video‐recorded and scored by an individual blinded to the genotype of the mouse. Data were analyzed using Ethovision XT video track system (Noldus Information Technology, Wageningen, The Netherlands).

### 2.4 Rotarod test

Motor performance and coordination were investigated by means of rotarod test in 16‐month‐old NURR1‐KO (male *n* = 6; female *n* = 9) and their WT littermates (male *n* = 6; female *n* = 5) and in 3–5‐month‐old female NURR1‐KO (*n* = 14) and their WT (*n* = 7) littermates. Mice were tested for three consecutive days. In each day, after a 2 min training session at a constant speed (4 rpm), the mice received three test sessions (T) in which the rod (Mouse Rotarod, Ugo Basile Biological Research Apparatus, Comerio, Italy) accelerated continuously from 4 to 65 rpm over 350 s (Hoxha et al., 2013; Montarolo et al., 2019). The latency to fall off the rod was recorded and reported as the mean of the third trials of each of the 3 days.

### 2.5 DA isolation and measurement

DA concentration was measured in brain and in plasma of NURR1‐KO and WT littermates. Specifically, a group of 16‐month‐old NURR1‐KO (male *n* = 5; female *n* = 9) and their WT littermates (male *n* = 3; female *n* = 6), and a group of 3–5‐month‐old female NURR1‐KO (*n* = 5) and their WT littermates (*n* = 8) underwent DA measurement in brain. Likewise, a group of 16‐month‐old NURR1‐KO (male *n* = 5; female *n* = 13) and their WT littermates (male *n* = 3; female *n* = 4), and a group of 3–5‐month‐old female NURR1‐KO (*n* = 4) and their WT littermates (*n* = 6) underwent DA measurement in plasma. NURR1‐KO and WT littermate mice were euthanized by inhalation of isoflurane and brains were removed and blood was collected into EDTA tubes. The brains were rapidly frozen in 2‐methylbutane in dry ice. Blood samples were centrifuged 10 min at 3000G at 4°C. The plasma was removed and rapidly frozen in 2‐methylbutane in dry ice. Brains and plasma were stored at −80°C until use.

DA hydrochloride (analytical standard grade), formic acid (HPLC grade), and ammonium formate (HPLC grade) were purchased from Sigma‐Aldrich Corporation (Milan, Italy). Acetonitrile (HPLC grade) was purchased from VWR (Milan, Italy). HPLC‐grade water was produced by a Milli‐DI system coupled with a Synergy 185 system by Millipore (Milan, Italy). Previously weighted brain samples were immersed in liquid nitrogen, sonicated for 1 min, reconstituted in 1 ml of water, and sonicated for another min. The calibration curve of DA was established in the concentration range of 0.01–5.00 μg/ml. One hundred microliter plasma or brain samples were extracted by protein precipitation using 300 μl of freeze solution of formic acid 0.5%v/v in acetonitrile. Each sample was mixed for at least 15″ and then stored in freezer at −20°C for 15′ and later centrifuged at 4000 rpm for 10 min. The 250 μl of supernatant was transferred to an injection vial. Chromatographic separation was performed at 35°C, using a column oven, on a RP column (Atlantis T3 4.6 × 50 mm, 5 μm, Waters, USA). The gradient chromatographic elution was executed through solution reported in Table 1. The flow rate was set at 1 ml/min. DA plasma concentrations were reported as μg/ml, instead brain amount was converted in μg/mg of tissue weight. For DA quantification, the limit of detectable (LOD) and limit of quantification (LOQ) in chromatographic method for determining DA were optimized with mixing homogenous samples (brain or plasma).

**TABLE 1:** Gradient chromatographic elution

Time (min)	%v/v Water	%v/v Acetonitrile	%v/v C 100 mM ammonium formate, pH = 3.00 (with formic acid)
0.00	95	0	5
2.50	95	0	5
7.00	40	55	5
9.00	40	55	5
9.20	95	0	5
13.50	95	0	5

### 2.6 Systolic blood pressure and heart rate measurement

A group of 16‐month‐‐old NURR1‐KO (male *n* = 4; female *n* = 9) and their WT littermates (male *n* = 8; female *n* = 8), and a group of 3–5‐month‐old female NURR1‐KO (*n* = 9) and their WT littermates (*n* = 5) underwent systolic blood pressure and heart rate measurement. Blood pressure and heart rate were recorded using a noninvasive tail cuff‐based technique (BP‐2000 Series II, Blood Pressure Analysis System, Visitech Systems, Apex, NC). Prior to measurements, the animals were placed into the habituation room for 30 min to ensure mice adaptation to the procedure. Parameters were recorded in a proper environment (RT, lightning and noise‐free atmosphere). Conscious mice underwent 5‐cycle measurement of systolic blood pressure and heart rate. The mean value of the 5‐cycle measurement was reported.

### 2.7 Statistics

Normality of distribution and homogeneity of variances were assessed by the Shapiro–Wilk and Levene's test. Robust two‐way ANOVA for main effects and interactions (using Rfit package [Kloke & Mckean, 2012]) or two‐tailed unpaired *t* test were used to compare continuous data between groups, as appropriate. Statistical significance was considered at *p* values < 0.05. All analyses were carried out using R version 4.1.1 (https://www.r‐project.com).

## 3 RESULTS

### 3.1 Behavioral phenotype of old NURR1‐KO mice

**FIGURE 1:**
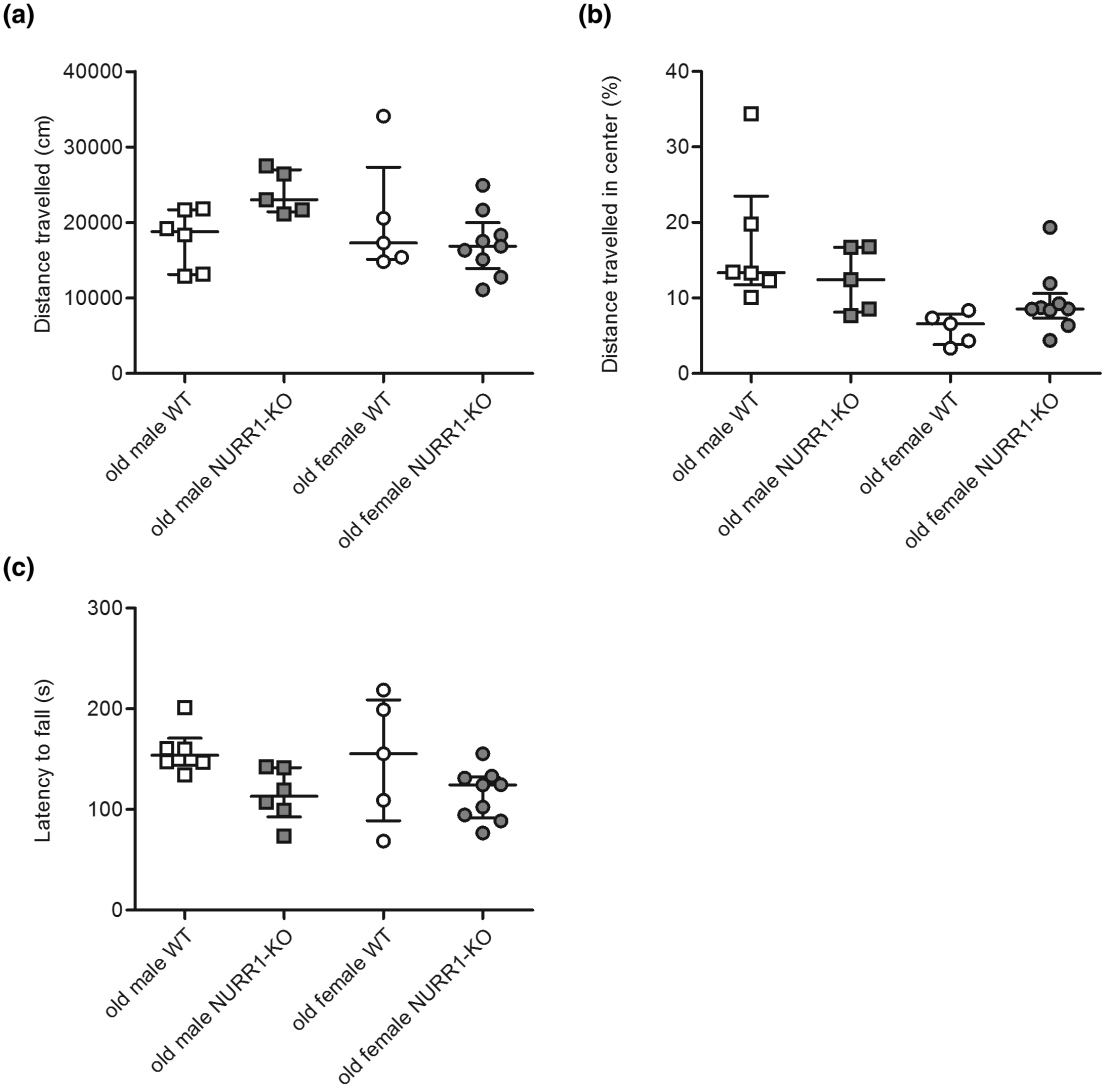
Behavioral phenotype of old NURR1‐KO mice. Both male (square) and female (circle) old WT (white) and NURR1‐KO (gray) mice were tested in the open field (OF) (a, b) and in the rotarod (c). Total distance traveled in the arena (a) and in the center of arena of the OF (b) are reported as centimeters (cm) and percentage of the distances traveled in the center versus the total distance, respectively. The latency to fall from the rotarod is reported as the mean of the third trials of each of the 3 days measured in seconds (s)(c). Line and bar indicate the median value and interquartile range. NURR1‐KO, NURR1 knockout; WT, wild‐type.

The OF and rotarod tests were performed on old mice to explore the effect of sex and NURR1 deletion on spontaneous locomotor activity and motor coordination. NURR1 deficiency affected the locomotion of male mice only. When placed in a novel OF, the longest total distance traveled was observed in male NURR1‐KO mice, while both groups of female mice showed no signs of hyperactivity (Figure 1a, robust two‐way ANOVA, sex: *F*(1,22) = 3.98, *p* = 0.059, genotype: *F*(1,22) = 2.40, *p* = 0.135, interaction: *F*(1,22) = 5.02, *p* = 0.035). The distance traveled in the center was lower in female mice, but was not affected by genotype (Figure 1b, robust two‐way ANOVA, sex: *F*(1,21) = 11.77, *p* = 0.002, genotype: *F*(1,21) = 0.02, *p* = 0.896, interaction: *F*(1,21) = 2.26, *p* = 0.148).

NURR1‐KO mice independently from their sex, showed a decreased latency to fall from the rotarod, suggesting a motor impairment (Figure 1c, robust two‐way ANOVA, sex: *F*(1,22) = 0.01, *p* = 0.926, genotype: *F*(1,22) = 5.31, *p* = 0.031, interaction, *F*(1,22) = 0.02, *p* = 0.899).

### 3.2 DA level in brain and plasma of old NURR1‐KO mice

**FIGURE 2:**
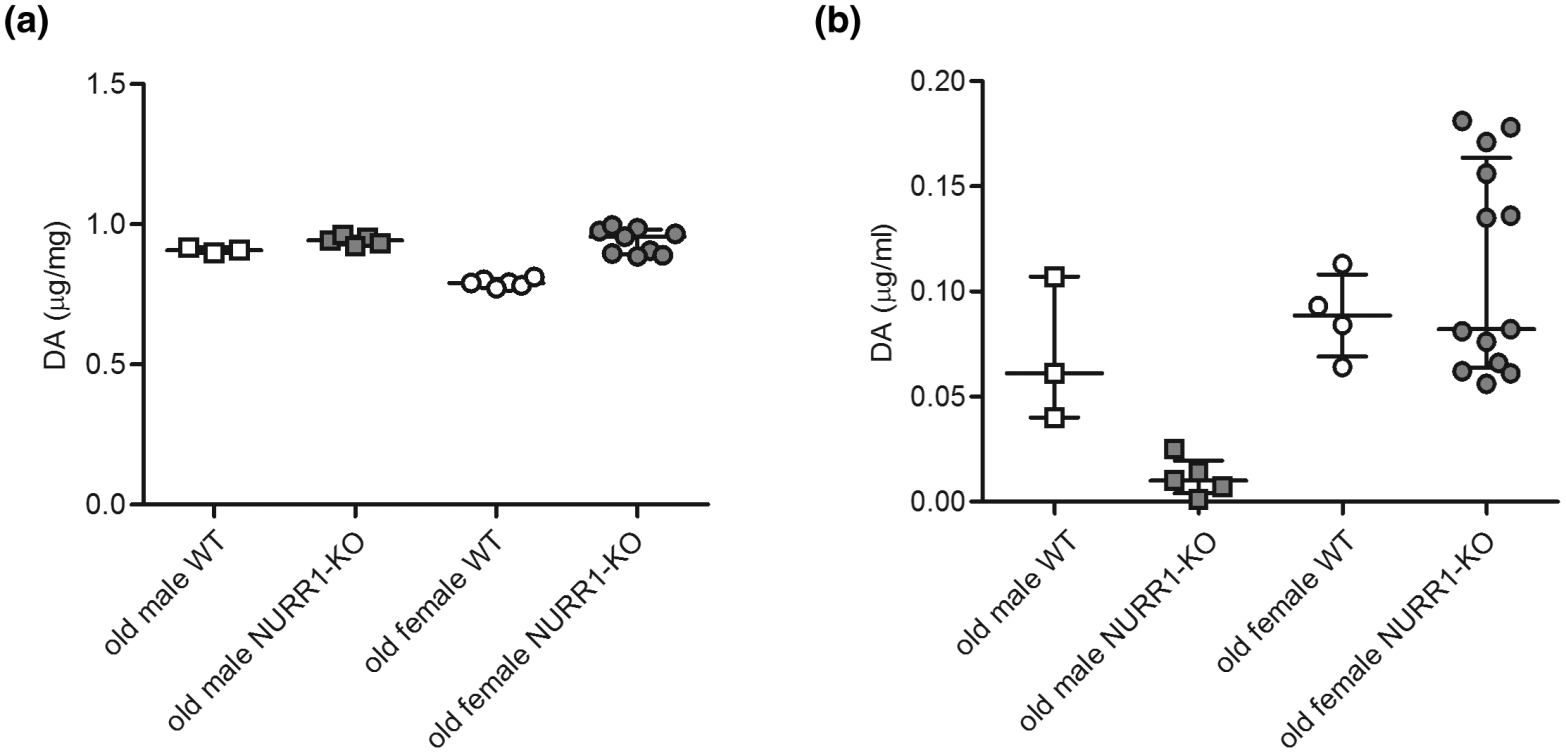
DA Level in brain and plasma of old NURR1‐KO mice. DA was measured in brain (a) and plasma (b) of both male (square) and female (circle) old WT (white) and NURR1‐KO (gray) mice. DA is reported as micrograms for milligram of brain tissue (μg/mg), and as micrograms for milliliter of plasma (μg/ml). Line and bar indicate the median value and interquartile range. NURR1‐KO, NURR1 knockout; WT, wild‐type.

DA level in brain of old mice was influenced by both their sex and genotype, as it was higher in male mice compared to females, and in NURR1‐KO compared to WT. In particular, the effect of genotype was more pronounced in female mice (Figure 2a, robust two‐way ANOVA, sex: *F*(1,19) = 19.68, *p* < 0.001, genotype: *F*(1,19) = 39.19, *p* < 0.001, interaction: *F*(1,19) = 16.19, *p* < 0.001). On the other hand, DA level in plasma was overall lower in old male mice, particularly in NURR1‐KO; however, DA level in female mice was not affected by genotype (Figure 2b, robust two‐way ANOVA, sex: *F*(1,19) = 12.27, *p* = 0.002, genotype: *F*(1,19) = 3.90, *p* = 0.062, interaction: *F*(1,19) = 3.41, *p* = 0.080).

### 3.3 Heart rate and systolic blood pressure of old NURR1‐KO mice

**FIGURE 3:**
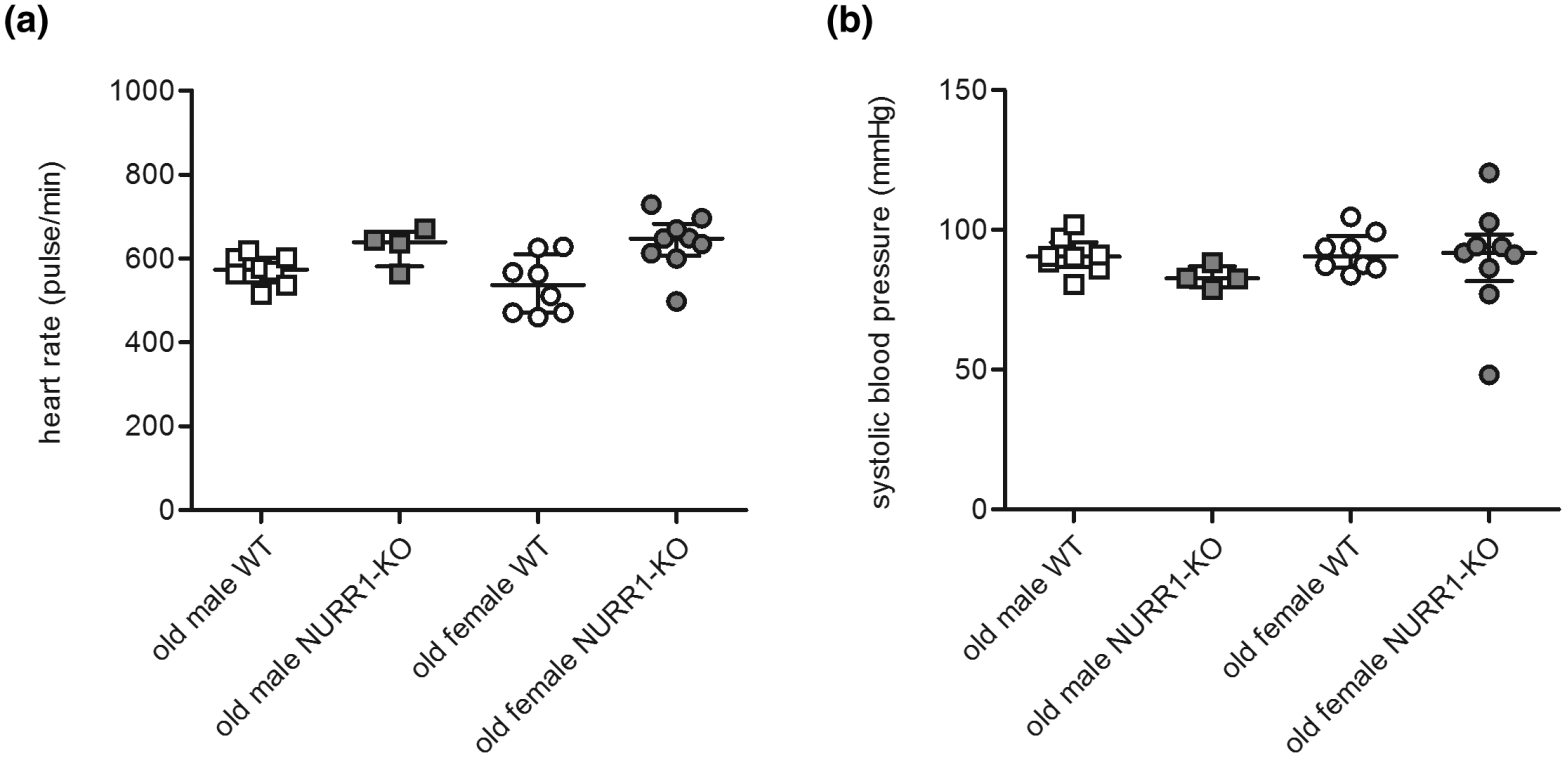
Heart rate and systolic blood pressure of old NURR1‐KO mice. Heart rate (a) and systolic blood pressure (b) measurement of male (square) and female (circle) old WT (white) and NURR1‐KO (gray) mice are reported as number of pulse/min and millimeters of mercury (mmHg), respectively. Line and bar indicate the median value and interquartile range. NURR1‐KO, NURR1 knockout; WT, wild‐type.

Heart rate and systolic blood pressure were evaluated based on the role exerted by peripheral DA level in the control of blood circulation (Ziegler et al., 1985). Heart rate was higher in old NURR1‐KO mice, independently from their sex (Figure 3a, robust two‐way ANOVA, sex: *F*(1,25) = 0.37, *p* = 0.546, genotype: *F*(1,25) = 12.05, *p* = 0.002, interaction: *F*(1,25) = 1.19, *p* = 0.285). On the other hand, systolic blood pressure was similar between groups (Figure 3b, robust two‐way ANOVA, sex: *F*(1,25) = 2.29, *p* = 0.142, genotype: *F*(1,25) = 1.54, *p* = 0.226, interaction: *F*(1,25) = 1.68, *p* = 0.206).

### 3.4 Characterization of young female NURR1‐KO mice

A group of young female mice was tested to evaluate if alterations in behavior, brain DA levels, and heart rate observed in old female NURR1‐KO mice are specifically related to aging. Young female NURR1‐KO mice showed no signs of hyperactivity and anxiety‐like behavior compared to their WT littermates, measured as total distance traveled (Figure S1a, unpaired Welch's *t* test, *t*[17.94] = −0.75, *p* = 0.465) and distance traveled in the center (Figure S1b, unpaired Welch's *t* test, *t*[15.17] = −2.07, *p* = 0.056) during the OF test. Interestingly, unlike the old ones, young female NURR1‐KO mice presented neither a motor impairment, as no significant differences emerged in the latency to fall compared with WT mice during rotarod test (Figure S1c, unpaired Welch's *t* test, *t*[7.30] = 0.60, *p* = 0.566), nor altered brain DA levels, as DA levels were similar between groups in both brain (Figure S2a, unpaired Welch's *t* test, *t*[7.73] = −1.14, *p* = 0.286) and plasma (Figure S2b, unpaired Welch's *t* test, *t*[6.25] = 1.65, *p* = 0.147). On the other hand, young female NURR1‐KO mice showed an increased heart rate in comparison to their WT littermates (Figure S2c, unpaired Welch's *t* test, *t*[6.26] = −3.65, *p* = 0.010), without alteration in systolic blood pressure (Figure S2d, unpaired Welch's *t* test, *t*[11.59] = −0.07, *p* = 0.947) as occurs in old female mice.

## 4 DISCUSSION

The transcription factor NURR1 regulates genes involved in the DA production and transport such as TH (the rate‐limiting enzyme involved in the catecholamine biosynthesis) and DAT1/VMAT2 (Kadkhodaei et al., 2009; Saucedo‐Cardenas et al., 1998; Smidt & Burbach, 2007). As a result, NURR1 is essential to the generation and functioning of the dopaminergic circuitry (Kadkhodaei et al., 2009; Saucedo‐Cardenas et al., 1998; Smidt & Burbach, 2007; Solomin et al., 1999; Zetterström et al., 1997) and its deregulation is involved in the development of DA‐associated brain disorders, such as PD (Jankovic et al., 2005; Montarolo et al., 2016).

To date, identifying a mouse model that comprehensively reproduces the complexity of PD still represent a challenge. For several years, the toxin‐induced mouse models, such as 6‐hydroxydopamine (6‐OHDA), and 1‐methyl‐4‐ phenyl‐1,2,3,6‐tetrahydropyridine (MPTP), have been the gold standard in PD research even though they do not fully recapitulate the symptomatology and the genetic of the disorder (Mustapha & Taib, 2021). However, genetic models of the disease have been suggested, such as behavioral models exhibiting impaired motor coordination related to alterations in genes involved in the DA signaling pathway (Blesa & Przedborski, 2014). Among these, the old NURR1‐KO mouse has been proposed as a genetic model of PD, since it showed altered locomotor activities at the rotarod test (Jiang et al., 2005). However, this finding was not confirmed in a subsequent study (Kummari et al., 2017). Based on these controversial results, here we explored the effects of the constitutive deletion of NURR1 on locomotor activity, motor coordination, brain and plasma DA levels, blood pressure and heart rate of old mice, to clarify whether the old NURR1‐KO mouse is a suitable behavioral model of PD. In addition, since male old NURR1‐KO mice only have been described in the literature (Jiang et al., 2005; Kummari et al., 2017), we focused on the potential effect of sex on the behavioral phenotype of NURR1‐KO mice.

In agreement with Jiang and colleagues (2005), but in disagreement with Kummari et al. (2017), we observed that the old NURR1‐KO mouse is characterized by motor impairment and thus represents a suitable model of PD. Notably, for the first time, we showed that this behavioral phenotype applies to both old males and females and is age related, since neither young male (Montarolo et al., 2019) nor young female NURR1‐KO mice are affected by motor impairment. Besides shared similarities, including an increase in brain DA level and heart rate found in both male and female NURR1‐KO mice, we also observed a sex‐specific behavior. Male old NURR1‐KO mice only have been shown to be hyperactive, as already reported for the young ones (Eells et al., 2002; Jiang et al., 2005; Montarolo et al., 2019; Rojas et al., 2007; Vuillermot et al., 2011, 2012).

Despite the low sample size that could have limited the power of the study, our study provides evidence that the old NURR1‐deficient mouse may be a satisfactory model to study behavioral phenotype characteristics of PD and to test the clinical efficacy of potential therapeutic agents. Further studies in simplified systems will be necessary to dissect the mechanism underlying these observations.

## AUTHOR CONTRIBUTIONS

All authors read and approved the manuscript. *Conceptualization,* M.F. and B.A.; *Methodology,* M.F., C.F., H.E., and D.F.S; *Formal Analysis,* M.F., M.S., C.F., and H.E.; *Investigation,* M.F., C.F., A.S., and H.E.; *Resources,* C.M.A. and B.A; *Writing – Original Draft,* M.F.; *Writing – Review & Editing,* M.F., M.S., C.F., A.S., D.F.S., H.E., T.F., and B.A.; *Visualization,* M.F., M.S., C.F., A.S., D.F.S., H.E., T.F., C.M.A., and B.A.; *Supervision,* D.F.S., T.F., C.M.A., and B.A.; *Funding Acquisition,* B.A.

## CONFLICT OF INTEREST

The authors declare no conflict of interest.

## Supporting information


**FIGURE S1** Behavioral phenotype of young female NURR1‐KO mice. Both WT (white) and NURR1‐KO (gray) young female mice were tested in the open field (OF) (a, b) and rotarod (c). Total distance traveled in the arena (a) and in the center of arena of OF (b) are reported as centimeters (cm) and percentage of the distances traveled in the center versus the total distance, respectively. The latency to fall from the rotarod is reported as the mean of the third trials of each of the 3 days measured in s (c). Line and bars indicate the median value and interquartile range. Unpaired Welch's *t* test. NURR1‐KO, NURR1 knockout; WT, wild‐type

**FIGURE S2** Brain and plasma DA level, heart rate, and systolic blood pressure of young female NURR1‐KO mice. (a, b) DA was measured in brain (a) and in plasma (b) of both young female WT (white) and NURR1‐KO (gray) mice. DA is reported as micrograms for milligram of brain tissue (μg/mg), and as micrograms for milliliter of plasma (μg/ml). Line indicates the median value. (c, d)heart rate (c) and systolic blood pressure (d) measurement of both young female WT (white) and NURR1‐KO (gray) mice are reported as number of pulse/min and millimeters of mercury (mmHg), respectively. Line and bars indicate the median value and interquartile range. Unpaired Welch's *t* test, *t*(6.26) = −3.65, *p* = 0.010. NURR1‐KO, NURR1 knockout; WT, wild‐type

## Data Availability

The data that support the findings of this study are available from the corresponding author upon reasonable request.
