# Supplementary figures and images for: NURR1‐deficient mice have age‐ and sex‐specific behavioral phenotypes
Source: J Neurosci Res. 2022 May 20;100(9):1747–54. doi: 10.1002/jnr.25067 (PMC9539971; doi:10.1002/jnr.25067)

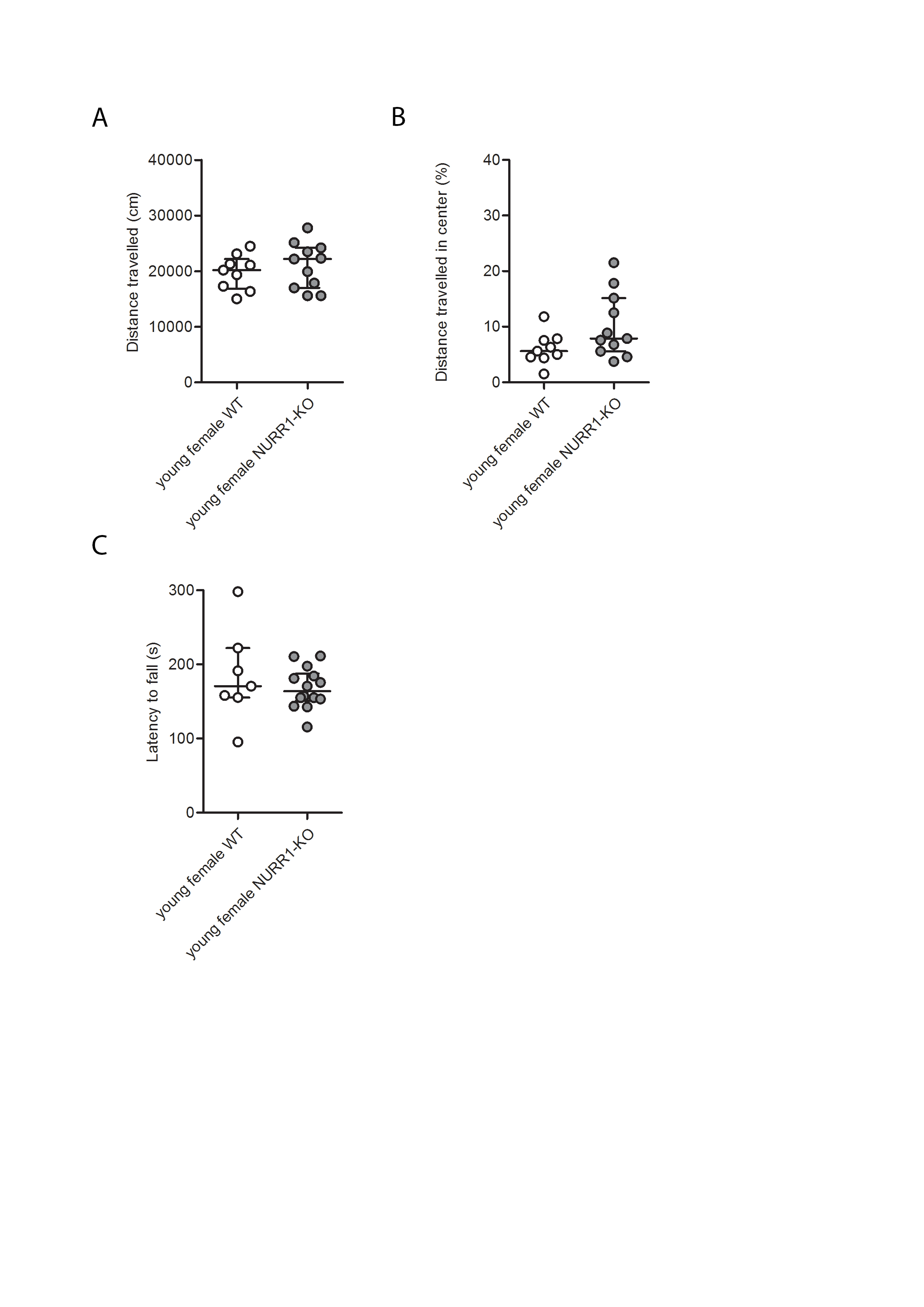

Supplement: Supplementary file 1 — FIGURE S1 Behavioral phenotype of young female NURR1‐KO mice. Both WT (white) and NURR1‐KO (gray) young female mice were tested in the open field (OF) (a, b) and rotarod (c). Total distance traveled in the arena (a) and in the center of arena of OF (b) are reported as centimeters (cm) and percentage of the distances traveled in the center versus the total distance, respectively. The latency to fall from the rotarod is reported as the mean of the third trials of each of the 3 days measured in s (c). Line and bars indicate the median value and interquartile range. Unpaired Welch's t test. NURR1‐KO, NURR1 knockout; WT, wild‐type [file JNR-100-1747-s001.tif]

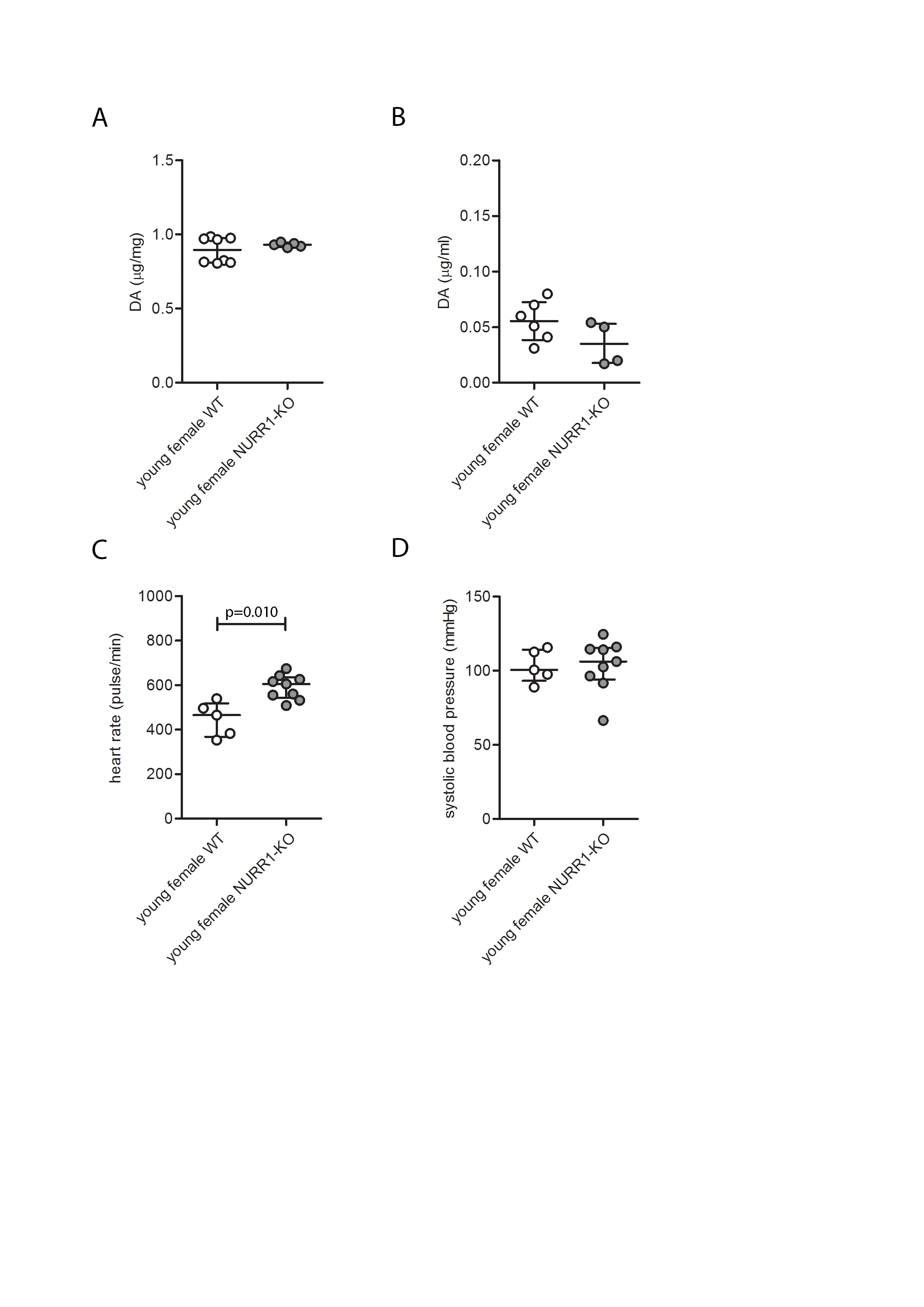

Supplement: Supplementary file 2 — FIGURE S2 Brain and plasma DA level, heart rate, and systolic blood pressure of young female NURR1‐KO mice. (a, b) DA was measured in brain (a) and in plasma (b) of both young female WT (white) and NURR1‐KO (gray) mice. DA is reported as micrograms for milligram of brain tissue (μg/mg), and as micrograms for milliliter of plasma (μg/ml). Line indicates the median value. (c, d)heart rate (c) and systolic blood pressure (d) measurement of both young female WT (white) and NURR1‐KO (gray) mice are reported as number of pulse/min and millimeters of mercury (mmHg), respectively. Line and bars indicate the median value and interquartile range. Unpaired Welch's t test, t(6.26) = −3.65, p = 0.010. NURR1‐KO, NURR1 knockout; WT, wild‐type [file JNR-100-1747-s002.tif]
